# Developing cardiac biomechanical models beyond the clinic: modeling stressors of daily life

**DOI:** 10.1007/s10237-025-01982-3

**Published:** 2025-07-10

**Authors:** Alexandre Lewalle, Tiffany M. G. Baptiste, Rosie K. Barrows, Ludovica Cicci, Cesare Corrado, Angela W. C. Lee, Cristobal Rodero, José Alonso Solís-Lemus, Marina Strocchi, Steven A. Niederer

**Affiliations:** 1 Cardiac Electromechanics Research Group (CEMRG), National Heart and Lung Institute (NHLI), Faculty of Medicine, Imperial College London, London, UK; 2 Present Address: School of Biomedical Engineering and Imaging Sciences, King’s College London, London, United Kingdom; 3 Turing Research and Innovation Cluster in Digital Twins, The Alan Turing Institute, London, United Kingdom

**Keywords:** Cardiac modeling, Computational simulation, Digital twins, Clinical translation

## Abstract

There is growing motivation to exploit computational biomechanical modeling of the heart as a predictive tool to support clinical diagnoses and therapies. Existing patient-specific cardiac models often rely on data collected under highly standardized conditions in hospitals. However, disease progression and therapy responses often depend on stressors, encountered in daily life, that cannot be captured in a traditional clinical setting. To achieve clinical translation, existing modeling frameworks must be refined and extended to include such influences. The “digital twin” concept, in which models of specific systems are continually updated with new data, is a promising avenue for integrating and interpreting these data streams. However, this endeavor calls for novel approaches to model development and data acquisition and integration. We review modeling approaches addressing specific stressor types (caffeine, exercise, sex-dependent factors, sleep, the environment) to identify knowledge gaps, assess emerging technical challenges, and suggest potential model developments to extend the scope and reach of biomedical cardiac simulations.

**Graphical abstract:**

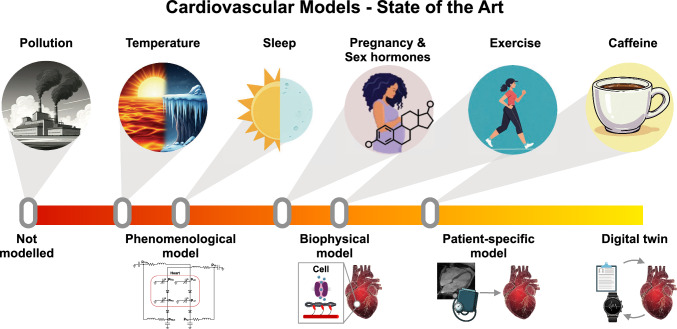

## Introduction

Biomechanical models of the heart have become invaluable tools for building an integrated understanding of cardiac function (Trayanova and Rice 2011; Trayanova 2011; Niederer et al. 2019; Wang et al. 2015). Typically, they encompass the anatomy, the passive and active material properties, and the external mechanical loading on the heart. They are intrinsically multi-scale, linking cellular electrophysiology and myocardial contraction through to whole-heart and circulation function. Model implementations range from lumped-parameter systems of ordinary differential equations through to full three-dimensional anatomical multiphysics coupled systems of partial differential equations. Through the computational solution, simulation, and analysis of such cardiac biomechanics models, we integrate disparate experimental and clinical data within physiology- and physics-constrained frameworks to investigate interdependences and interactions in silico that may otherwise be inaccessible experimentally or clinically.

There is an increasing motivation to harness the predictive power of computational simulations in clinical settings to support the monitoring (Moss et al. 2021), diagnosis, and treatment of various heart conditions (Nguyen et al. 2020; Gerach et al. 2021, 2022, 2023; Dupuis et al. 2018; Roel 2023). However, this ambition encounters significant hurdles. Existing patient-specific cardiac models often rely on imaging or diagnostic data that are collected under highly standardized conditions while patients are at rest in the hospital. Yet, disease progression and therapy response are often significantly impacted by multiple factors dictated by life outside healthcare centers. Such factors can include the daily life cycle (physical exertion, sleep, mental stress), environmental conditions (temperature variations and exposure to pollution), and other influences derived from other body systems (e.g., hormonal response or nutrition). These factors can modulate the baseline physiology of the cardiovascular system on both acute and chronic time scales. In conventional cardiac models, which focus on describing generic cardiac physiology, they are effectively averaged out or altogether ignored. Despite substantial recent developments in biomechanical modeling techniques, this challenge constitutes a fundamental and novel obstacle to practical application development. In summary, future progress will require addressing challenges on two related fronts. On the one hand, modeling frameworks must accommodate the breadth of phenomena that influence cardiovascular physiology, including those not considered in conventional clinical assessments. On the other hand, once these effects are formally implemented, they must be characterized systematically and quantitatively using appropriate data sources and analysis methods that also extend beyond conventional clinical protocols.

Delivering the so-called five rights of patient care [the right patient, the right drug, the right time, the right dose, and the right route (Grissinger 2010)] requires the continual and systematic monitoring of a patient’s condition. In this perspective, the growing availability of data (in particular following the advent of wearable measurement devices) opens opportunities for continuously monitoring patients in the community under a much wider range of conditions, physiological activity, and stressors than those normally experienced in the hospital. The adoption of the “digital twin” (DT) concept from engineering will be essential to integrate and interpret continuous input data streams.

The National Academies of Sciences, Engineering, and Medicine (NASEM) define a DT as “a set of virtual information constructs that mimics the structure, context, and behavior of a natural, engineered, or social system (or system-of-systems), is dynamically updated with data from its physical twin, has a predictive capability, and informs decisions that realize value” (NASEM 2024). The bidirectional interaction between the virtual and the physical objects is thus central to the underlying principle of DTs, which are continually updated with newly available data and, simultaneously, compute information that can assist in real-world decision making. DTs can be used to track changes in a physical object and optimize its function and efficiency. There is therefore a clear appeal to extend this concept to achieve a cardiac DT (CDT). However, many present-day cardiac biomechanical modeling efforts focus on capturing the physiological behavior at a fixed moment in time, giving little attention to its long-term variability. The challenge stems both from the extreme complexity of the cardiac physiology and from the intrinsic difficulty to capture external influences and assign them systematically to specific model properties. As discussed later, “growth and remodeling” (G&R) models may provide a promising avenue to address this.

**Fig. 1:**
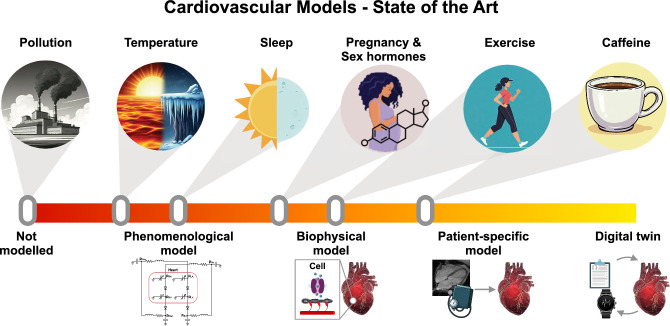
Schematic graphical representation of the present state of the biomechanical modeling of cardiovascular stressors described in this review. The general classes of stressors, depicted by the icons in the upper row, are placed on the central axis, which represents the degree of CDT development. Heuristically, the stages of this development are represented pictorially using the icons in the lower row, representing (1) phenomenological modeling, which quantitatively predicts observable behavior without reference to specific biophysical mechanisms; (2) biophysical modeling, which invokes specific mechanisms calibrated using clinical and/or experimental measurements; (3) patient-specific modeling, which accounts for detailed patient-wise variability; and (4) the “complete” cardiac digital twin, taken as a utopic end goal. (The figure was partly generated using Gemini. All the images used in this figure were generated between November 8, 2024 and April 30, 2025)

The aim of this review is to identify some of the hurdles facing the realization of CDTs and to propose realistic solution pathways. We consider a selection of common external stressors known to impact cardiovascular function: caffeine consumption, physical exertion, sex-dependent factors, sleep, and environmental influences. Our general assessment of the varying extent of modeling development in each case is summarized in Figure 1. For each category, we review key biomechanical modeling studies reported in the literature that have sought to account for their impact on cardiovascular function. This provides a perspective for identifying new directions for model development and refinement going forward.

## Caffeine

Caffeine, a common ingredient present in many people’s daily nutrition, has a well-documented impact on cardiac function. Its dose-dependent effects on sympathetic activation, intracellular calcium dynamics, adenosine receptors, and susceptibility to arrhythmia have been extensively reviewed (Zulli et al. 2016; Turnbull et al. 2017; Voskoboinik et al. 2018). A moderate dose of caffeine increases the sinus rate and enhances the cytosolic calcium concentration by increasing the probability of the sarcoplasmic reticulum (SR) release channels (RyR) and suppressing SR reuptake. A comprehensive understanding of the clinical impact of caffeine consumption must therefore consider, on the one hand, its direct biochemical effect at the cellular level. On the other hand, a pharmacokinetic/pharmacodynamic (PKPD) analysis is equally necessary to determine the exposure profile of the drug within the blood circulation following oral ingestion. The inherent variability of both these aspects—between individuals and also over time in a given individual—make this cardiac stressor well suited to analysis within a CDT framework.
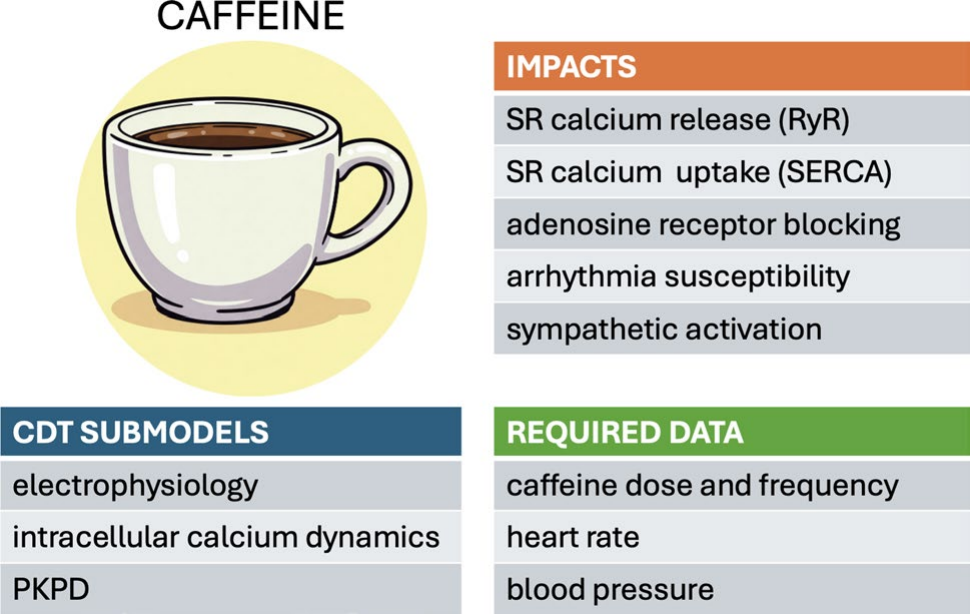


Computational models of caffeine impact at the cellular level generally involve ODE-based electrophysiological models of the calcium cycling dynamics within single myocytes. These models are typically calibrated using animal experimental models. To identify the most likely mechanism of action, Yaniv et al. considered rabbit pacemaker cells and compared the predictions of different models to experimental measurements (Kurata et al. 2002; Maltsev and Lakatta 2009; Severi et al. 2012; Yaniv et al. 2013). They concluded that a low caffeine concentration is likely to affect the sensitivity of RyR activation by lumenal SR calcium (Yaniv et al. 2013). The pacemaker cell rate was also partially accelerated via a change in SR calcium loading due to accelerated SR calcium pumping. Using a mouse myocyte electrophysiological model, Morotti et al. reproduced the calcium efflux dynamics during a caffeine-induced calcium transient by tuning the SR uptake and the sodium/calcium exchanger (NCX) (Morotti et al. 2014). Despite general agreement on the overall mechanisms involved, the finer details of calcium flux balance are currently still being debated (Eisner et al. 2017; Terrar 2024; Eisner et al. 2024).

Physiologically based pharmacokinetic (PBPK) models aim to predict the concentration profile of a drug or metabolite at chosen body locations over time. This involves the consideration of the processes of absorption, distribution, metabolism, and excretion of the substance at the systemic level. For example, a PBPK study by Darakjian et al. related caffeine consumption and caffeine plasma level in pregnant women (Darakjian and Kaddoumi 2019). The PBPK models were validated by comparing the virtual population data with clinical data. Whereas conventional medical practice generally adopts a one-size-fits-all approach based on a so-called “average patient”, the importance of accommodating for patient-specific factors is increasingly recognized. Fendt et al. present a PBPK model of caffeine, personalized using age, height, weight, sex, and physiological/metabolic data (Fendt et al. 2021). Model personalization substantially improved the predictive accuracy relative to that of an “average” baseline model. These developments open promising perspectives for predicting personalized pharmacodynamic responses to caffeine and other drug treatments generally within the CDT context.

## Physical exercise

Physical exertion affects multiple body systems on both short and long time scales. Various computational biomechanical modeling studies have explored the effects of exercise in healthy and diseased patients, probing the complex interplay between the cardiovascular system, the circulation, the respiratory system, gas exchange and the sympathetic and parasympathetic nervous systems. Various aspects of exercise could be further incorporated into models through modifications to the autonomic nervous system, hormonal changes which could in turn effect changes to parameters describing vascular resistance and cardiac contractility, as well as blood pressure regulation, heart rate, cardiac output, blood-flow distribution, and vascular compliance. Simulating the effects of changes in exercise patterns in longitudinal studies would help to determine the contribution of these factors to cardiac function over time.
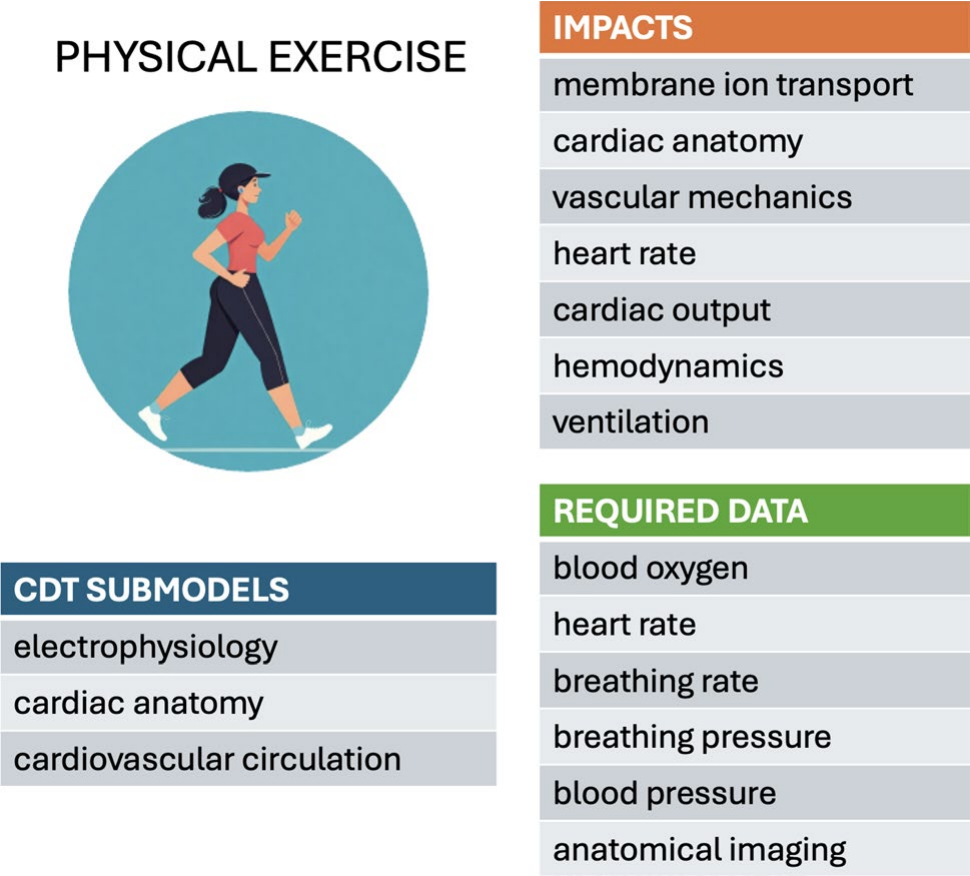


Existing models typically assume a closed-loop representation of the cardiovascular system (CircAdapt), including the pulsating heart, the pulmonary and systemic circulation, a vascular bed model, local metabolic vasodilation, and mechanical effects of contraction. To allow the tractable solution of such multifaceted systems, the short-term or instantaneous response to exercise is often simulated using lumped-parameter models (where separate components are combined into a point-like entity) (Granegger et al. 2018; Kaye et al. 2021; Lumens et al. 2019; Elisa and Ursino 2002; Olsen et al. 2021; Pearce and Kim 2023; Sarmiento et al. 2021; Sturgess et al. 2024). The phenomenological nature of such models limits their scope for capturing an individual’s detailed mechanisms of pathology and functional and electrophysiological characteristics. The use of whole-organ finite-element models, while providing more mechanistic insights, comes at a significantly greater computational cost (Govindarajan et al. 2024; Yao et al. 2022). On the other hand, the long-term evolution of the cardiac system in response to exercise has been modeled by combining finite-element modeling and Bayesian optimization of growth models (Yiling et al. 2021).

Notwithstanding the generic insights provided by these models, the cardiovascular system response to physical exercise inevitably remains strongly dependent on the individual’s condition. Personalized models would therefore enable simulations of a given patient’s behavior to customize treatment options.

### Modeling short-term effects

The release of epinephrine and norepinephrine by the body during exercise stimulates beta-adrenergic receptors, which in turn increases calcium influx and uptake in cardiomyocytes. In this context, Lyon et al. (2020) modeled the dynamic interaction between the mechanical and electrophysiological subsystems via a bidirectional coupling of the O’Hara/Rudy human ventricular electrophysiological cell model with a mechanical model of sarcomere mechanics. The excitation-contraction coupling was simulated by inputting the intracellular calcium predicted by the electrophysiology model into the mechanical model to generate force. The dynamics of calcium buffering by troponin in the mechanical model was then fed back into the electrophysiological model. Beta-adrenergic stimulation was simulated in the electrophysiology model by increasing specific ion-channel currents and by decreasing the troponin/calcium binding affinity. In addition to the effects in the electrophysiological cell model via beta-adrenergic stimulation, exercise also increases the preload on the heart, which can be modeled by changing the sarcomere length. These combined effects increased force generation and quickened relaxation with lower calcium levels during diastole.

Cardiovascular disease is generally associated with impaired exercise capacity. Lumens et al. (2019) found that right-ventricular (RV) electromechanical dyssynchrony has a greater impact on exercise capacity than pulmonary regurgitation in patients after repaired tetralogy of Fallot (rToF). Their modeling approach used a series of ordinary differential equations to represent the systemic and pulmonary circulations in terms of resistance to flow, active contractile atria and ventricles, compliant blood vessels, and valves with inertia. Exercise was simulated by increasing the heart rate (HR) and adjusting the homeostatic pressure-flow regulation to increase the target cardiac output (CO) (Lumens et al. 2019). By using lumped-parameter models, Olsen et al. (2021) studied patients with pulmonary arterial hypertension, simulating exercise by increasing the HR and force generation, coupled with a reduction in myocardial activation time and vena cava resistance. They thus showed that exercise capacity is limited by RV function. Using a similar electric-circuit analogue of the cardiovascular system, Kaye et al. (2021) investigated the hemodynamic mechanisms for reduced exercise capacity in HFpEF (heart failure with preserved ejection fraction) patients and found the estimated stressed blood volume to be significantly higher in this population.

The baroreflex is an acute regulatory system that maintains homeostasis by stabilizing blood pressure via changes in muscle contractility, the HR, and the total peripheral resistance. During acute exercise, the blood pressure rises to increase the cardiac output and ensure sufficient blood supply to the skeletal muscles. The 1998 model by Ursino was the first to describe the interaction between the baroreflex and the pumping heart with pulsatile pressure (Ursino 1998). By using an electric-circuit analogy, treating the heart as a voltage source, this model elucidated the relationship between the pressures (voltage), resistance, and compliance with the blood flow (current) through the pulmonary and systemic circulations and the cardiovascular control system. This lumped-parameter model was further developed by Magosso and Ursino to incorporate the effects of dynamic aerobic exercise (Elisa and Ursino 2002). This involved representing the contracting heart as a time-varying voltage source, allowing for the beat frequency and contractile force (voltage amplitude) to be adjusted, thereby increasing the complexity of the vascular system, as well as incorporating the autonomic nervous system and baroreceptor reflex to achieve a more realistic physiological representation. To simulate the effects of exercise, they increased the HR and CO targets; they applied vasodilation to enhance blood flow to the active skeletal muscle vascular beds and vasoconstriction to reduce flow to the other vascular beds; the baroreflex control circuit comprised a feedback system. These changes were shown to counteract the effects of exercise on blood pressure and maintain it at a specific level.

The impacts of exercise extend beyond the cardiovascular system to include the respiratory system. Oxygen consumption provides a measure of metabolic demand in the body. The integrated cardiovascular and respiratory model of exercise by Sarmiento et al. amended the Magasso/Ursino model by including respiratory mechanics, respiratory control mechanisms, and a gas exchange system (Sarmiento et al. 2021). The effects of exercise on the healthy respiratory system was simulated by increasing oxygen consumption, carbon dioxide production, the breathing rate and depth, in addition to the changes in the cardiovascular system discussed above. By incorporating these mechanisms, the model was able to simulate the physiological changes that occur during exercise, including increased CO, increased ventilation, and altered blood flow distribution.

Potential treatment options and the predicted impact of treatments on patients under exercise conditions have also been investigated using computational cardiac models. Granegger et al. used a lumped parameter model to investigate the effects of rotary pumps in Fontan (univentricular heart) patients (Granegger et al. 2018). This model exploited the Ursino baroreflex model to simulate the effects of exercise with adjustments to the HR, systemic vascular resistance, contractility of the systemic ventricle, and mean circulatory filling pressure. The results suggested that an external pump could provide adequate support to a patient at rest but may prove detrimental and impair venous return under exercise conditions. Thus, control of the pump speed based on pressure changes was shown to be essential for improving cardiovascular function in Fontan patients.

Model validation constitutes an ongoing challenge. Some of the above studies report using adaptations of previously validated models describing the cardiovascular system, respiratory mechanics, gas exchange and hemodynamics, validated against experimental exercise performance data and other existing models in “virtual experiments” (Kaye et al. 2021; Sarmiento et al. 2021; Pearce and Kim 2023; Elisa and Ursino 2002). Lyon et al. validated their cellular model by comparing simulated calcium and force dynamics under $\beta$-adrenergic stimulation and stretch with in vitro cardiomyocyte experiments, confirming mechanistic accuracy (Lyon et al. 2020). The study of Fan et al. was validated by comparing simulated exercise-induced myocardial growth with longitudinal imaging data, assessing the performance of the Bayesian optimization, and ensuring biomechanical plausibility (Yiling et al. 2021).

However, validation can be intrinsically problematic. As argued by Lumens et al. (2019), “predictions afforded by modeling could not and cannot be entirely validated”, in particular owing to “the cross-sectional retrospective observational design”. Lyon et al. (2020), call for validation in future studies, “including the specific weighting of each risk factor and the addition of new risk factors as they are identified.”

### Modeling long-term effects

Sustained exercise drives functional and structural changes in the heart on much longer time scales than the acute responses described above. Exercise promotes changes in the left ventricle (LV) cavity and wall thickness, leading to physiological cardiac hypertrophy. Such long-term remodeling depends on the type of exercise: static exercises such as weightlifting promotes concentric hypertrophy (where the LV wall thickens and the LV cavity size decreases), whereas dynamic sports such as long-distance running predispose to eccentric cardiac remodeling (where the LV cavity enlarges).

To investigate these effects, Fan et al. modeled exercise-induced LV growth in swine that had undergone treadmill exercise training over a three-month period (Yiling et al. 2021). Finite-element (FE) models, generated from magnetic resonance images captured at discrete time points over the study period, were subjected to boundary conditions describing the corresponding pericardial constraints and hemodynamic loading. A transversely isotropic growth law was then applied to the initial model, describing longitudinal growth along the fiber directions and transverse growth along the sheet and normal directions. The optimal growth-model parameters were hence determined using Bayesian statistics. This innovative approach, combining the power of FE analysis with the efficiency of Bayesian optimization, has the potential to enable predictions of dynamic cardiac adaptation to pathologies or treatments over time. However, several challenges remain to be addressed to make this method usable in routine clinical practice. These challenges include the high computational costs, limited accuracy arising from the inevitable simplification of the cardiac anatomy, physiology, and growth mechanisms, and the paucity of high-quality longitudinal data required to generate accurate personalized models. Additional biological factors (hormonal, genetic, or relating to cell type) may yet need to be adequately accounted for.

Model validation is an intrinsic challenge of long-term growth modeling. As explained by Yiling et al. (2021), this requires, firstly, large amounts of experimental data, in addition to those used for model calibration. As a further complication, it is difficult to ascertain whether an observed evolution is exercise-induced rather than arising from overall body mass variation. The authors advocate the development of the validation methodology, in particular by exploiting histological measurements.

## Sex-based factors

Advances in clinical and pharmacological treatments increasingly highlight the need to include sex as a relevant factor when evaluating drug safety and optimizing therapeutic dose regimens. Significant differences in drug impact have been observed between male and female patients. This calls for a systematic incorporation of sex-dependent variations into electromechanical simulations. Furthermore, female cardiac and vascular anatomies differ from men in terms of both size and morphology (Pierre et al. 2022). In other words, the average female heart is not simply a scaled-down version of the male heart. Image-based models are inherently sex-specific when generated from imaging datasets collected from both male and female subjects. However, women are often under-represented in clinical studies about cardiovascular diseases, which can result in imbalanced populations of virtual models. In addition, other features such as fiber-orientation and material properties are included in existing models without discriminating between male and female. This assumption stems from a lack of relevant clinical and experimental data. When data become available, it will become possible to include these nuances in computational frameworks. Thus, electromechanical and fluid-dynamical modeling in the context of sex-specific cardiovascular health (ranging from cardiac remodeling induced by pregnancy, to increased risk of aortic aneurysm rupture) is still in its infancy.
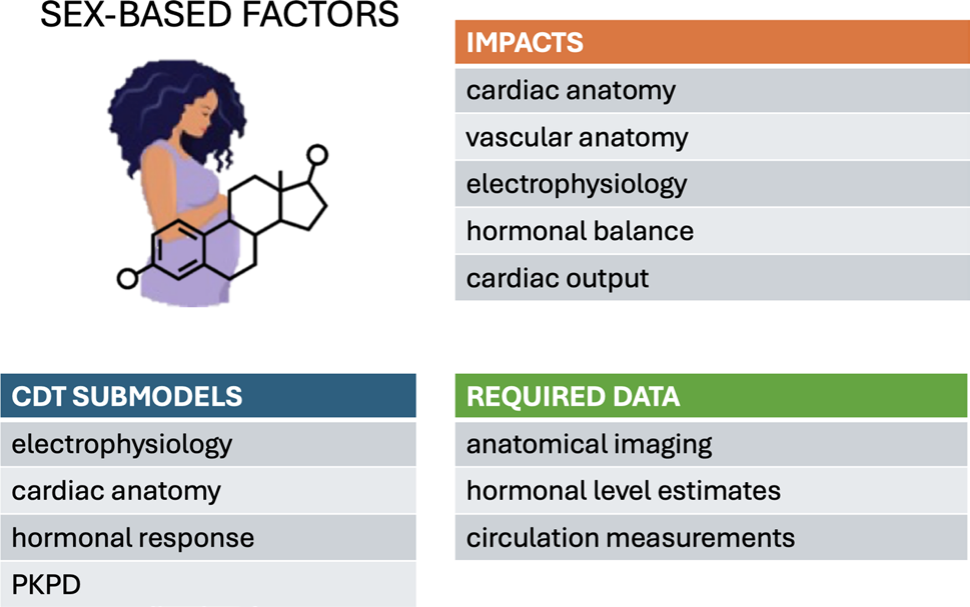


### Sex-specific models

Commonly used human single-cell electrophysiological models of the action potential typically neglect the sex of the subjects used for parameter calibration. Consequently, the models are assumed to represent the generic ventricular myocardium. Sex-dependent differences at the genome-level have only recently been considered within the context of cardiac modeling, typically by modifying the amplitude of key ion-channel currents, based on experimental studies on either human or animal hearts (Gaborit et al. 2010). Such sex-specific model parameterizations have been used to investigate the individual contribution of genomic differences to the cardiac electrophysiology and predict arrhythmia susceptibility (Yang et al. 2010; Yang and Clancy 2012; Yang et al. 2017; Verkerk et al. 2005, 2007; Cieniawa et al. 2013; Fogli et al. 2021). Yang et al. accounted for the acute effects of sex steroid hormone concentrations, which are incorporated on specific ion channels (Yang et al. 2010; Yang and Clancy 2012; Yang et al. 2017). They validated their predictions against either experimental measurements derived from guinea-pig ventricular myocytes incubated with human physiological concentrations of sex hormones or surrogate markers of arrhythmia risk. Sex-based cardiomyocyte models, often coupled with pharmacokinetics models, have been employed to investigate the effects of genetic background on drug response, and hence to predict the physiological effects of particular drug treatments (Varshneya et al. 2021; TeBay et al. 2022; Llopis-Lorente et al. 2023; Hellgren et al. 2023; Peirlinck et al. 2021). Sex-specific modulations of cardiac electrophysiology have also been investigated in models of the sinoatrial node (Doris et al. 2019) and atrial myocytes (Zhang et al. 2024). Understanding the effects of sex-specific ion-channel properties and the effect of sex hormones on ionic currents has therefore turned out to be essential for understanding differences in arrhythmia susceptibility between men and women. In this context, single-cell models can quantify these effects and provide mechanistic insight that would be difficult to gain through clinical measurements alone.

The importance of patient-specific anatomies is increasingly recognized in the context of heart failure development (Strocchi et al. 2020). However, most computational studies in the context of sex-related drug-induced arrhythmias have focused on single-cell models, neglecting the three-dimensional anatomy. Only a few recent studies employed 3D electrophysiological models to assess the role of sex-related phenotypes at the cellular (Llopis-Lorente et al. 2023; Gonzalez-Martin et al. 2023; Aguado-Sierra et al. 2024) or organ level (Lee et al. 2019) or both (Peirlinck et al. 2021) in cardiac electrical activity and arrhythmogenesis.

Evidence suggests that sex-specific cardiac models may have tangible benefits for understanding cardiovascular health risks more generally. Gao et al. (2020) observed that women’s blood vessels are smaller on average and also differ in terms of their curvature. Using computational simulations, they demonstrated that these anatomical characteristics also translated into functional differences, with aneurysms in women experiencing higher pressures and shear stresses, likely contributing to an increased risk of rupture. Although this model lacked a thorough validation of the model predictions, it still provides a potential mechanistic explanation of the higher risk of aneurysm rupture clinically observed in women compared to men. As that study shows, a thorough understanding of the overall cardiovascular system is thus likely to rely on a systematic sex-specific characterization of the cardiac subsystem.

### Pregnancy

Pregnancy is a complex and finely regulated process where the mother’s body adjusts to the growing fetus. In particular, the cardiovascular system adapts to support the mother and baby, leading to a 50% increase in the CO and circulating blood volume, accompanied by a drop in the systemic resistance by more than 30% (Pritchard 1965; Hunter and Robson 1992). Furthermore, estrogen and progesterone levels vary according to the gestational period (Tulchinsky et al. 1972), which also affects the cardiovascular system. Different types of computational models have investigated the consequences of these adaptations. Yoshida et al. used a rat-based cell-growth model to quantify the effects of estrogen, progesterone, and angiotensin II (which are also elevated during pregnancy) on myocyte remodeling (Yoshida et al. 2022). The model was calibrated using data collected during volume overload, infusion of angiotensin II, estrogen or progesterone in isolation, and was validated against data collected during a combination of these factors. The cell growth model was then coupled with a compartmental model to represent the rat’s circulatory system, linking hormonal changes to the cardiovascular response. The authors thus suggested that the early rise in progesterone in the first half of the pregnancy is an important contributor to heart growth during pregnancy.

Blood-flow modeling has been used to study arterial adaptation during pregnancy. Poppas et al. combined a 0D model of the arterial circulation with clinical data collected at different gestational stages to characterize pressure waveforms and the coupling of the heart to the circulatory system in healthy pregnancies (Poppas et al. 1997). While this model was not strictly validated, it highlighted the strength of even a simple computational framework for analyzing a rich clinical dataset. Sedaghati et al. combined a 1D blood-flow model with growth and remodeling to simulate the arterial tree and its adaptation to increased CO during healthy and pre-eclampsia pregnancies (Sedaghati and Gleason 2023). The model predictions over the gestational period were validated using literature data collected during healthy pregnancy, and early and late pre-eclampsia. That study showed that pre-existing higher arterial stiffness in the mother contributes to pre-eclampsia at a later gestational period.

### Pharmacological side effects

The cardiovascular system is indirectly affected by pathologies and disease treatments that do not primarily target the heart. For instance, the cardiotoxic nature of chemotherapies (often associated with sex-specific forms of cancer) calls for a refinement of existing models to better characterize and predict treatment outcomes. Assessing the cardiotoxicity of chemotherapies and their long-term consequences on the heart is fundamental for optimizing the life quality of breast cancer survivors. Pre-menopausal women diagnosed with breast cancer are often treated with anti-estrogen therapy. The cardioprotective effects of estrogens may imply severe consequences for cancer patients. Miller et al. combined magnetic resonance imaging with biomechanics modeling of the LV to quantify changes in wall elasticity following anti-estrogen cancer treatment (Miller et al. 2021). Local changes in the passive elasticity of the LV wall were quantified by using cardiac MRI frames recorded pre- and post-anti-estrogen treatment from pre-menopausal women with breast cancer. The results of the computational analysis suggested that anti-estrogen treatment causes myocardial stiffening, similar to ischemic damage. This result highlights the need to balance anti-cancer treatment with cardiotoxicity, especially in pre-menopausal women who would still benefit from the cardioprotective effects of estrogens. The modeling framework was validated in phantoms including a wide range of deformation patterns. However, as noted by the authors, more thorough validation to mimic the myocardium during passive filling is required to increase the trustworthiness of the model predictions.

In conclusion, sex-specific cardiac modeling is largely in its infancy. As our understanding of these specificities continues to grow, opportunities will arise for refining our understanding of pharmacological and hormonal treatments, including within the context of individuals undergoing gender transitioning.

## Sleep

The daily sleep-wake cycle regulates the body’s physiology via the circadian rhythm, hormone levels, metabolism, and cardiac function. During sleep, HR and blood pressure decrease and blood vessels dilate, increasing blood flow. Hormonal balance affecting blood pressure, HR, and HR variability (HRV), become disrupted by poor sleep quality, leading to hypertension, atherosclerosis and coronary artery diseases (Kanki et al. 2023; Cappuccio et al. 2011). Baroreflex sensitivity and function (described above in the context of exercise) also undergo changes during sleep. The lowering of the baroreflex set point, in conjunction with increased baroreflex sensitivity, leads to lower blood pressure during sleep. Sleep disorders have been found to impact cardiac health, with severe obstructive sleep apnea associated with increased risks of cardiovascular disease and mortality (Marin et al. 2005).
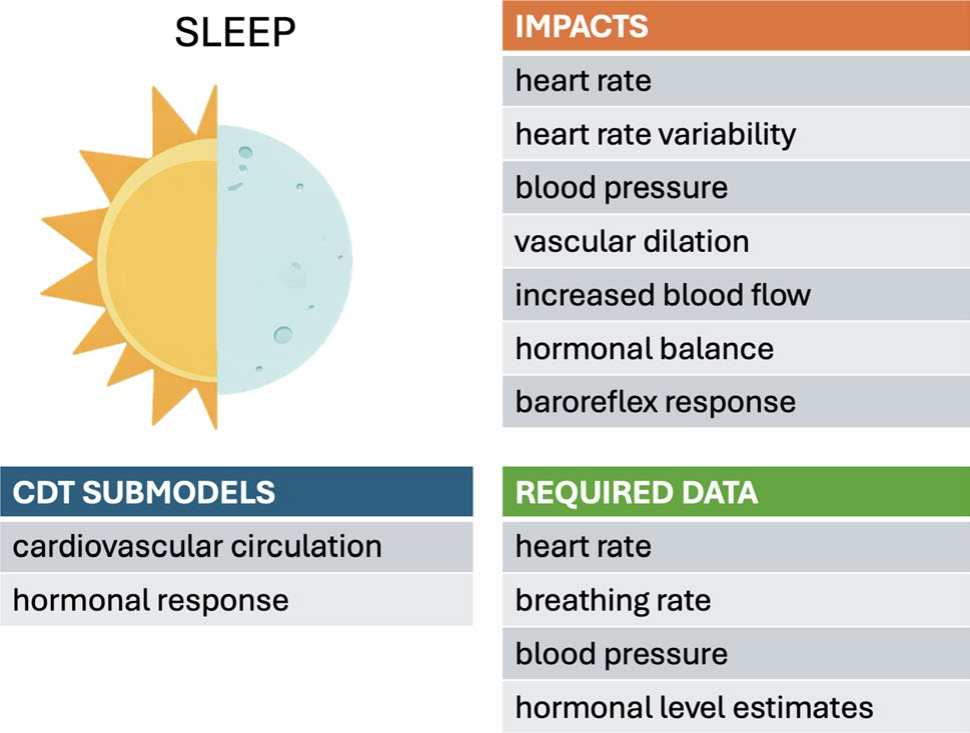


### Sleep-based models

Systematic models connecting sleep to cardiac functions remain scant. The simulation model by Cheng et al. integrates an Ursino-derived cardiovascular model with models of the respiratory system and neural control systems while including sleep-wake regulation (Cheng et al. 2010). Respiratory and cardiovascular control centers were modeled to regulate the breathing rate and depth, as well as the HR and blood pressure, necessary to maintain oxygen and carbon dioxide levels within prescribed ranges. The model was validated by assessing its ability to reproduce the baseline conditions of a general population under normal and various sleep states and under different interventions.

Different stages of sleep, so-called “non-rapid” and “rapid eye movement” (REM), were also included in the model to simulate corresponding changes in HR variability. The effects of sleep apnea and various treatment options were further explored with the addition of a metabolic subsystem (Cheng and Khoo 2012). This metabolic subsystem included glucose-insulin regulation and hormonal regulation, allowing an investigation of the metabolic consequences of obstructive sleep apnea and longer-term effects of disorders.

Further basic research is needed to establish quantitative relationships between key indicators of declining cardiovascular function (e.g., the ejection fraction, diastolic function, vascular stiffness) and observable parameters related to sleep. These relationships would inform how the model parameters are adjusted based on the tracked data. Sleep-associated parameters are likely to include sleep duration, quality (e.g., light, REM, and nonREM), and timing relative to the circadian rhythm, as well as the incidence of sleep disorders. Wearable technology can be a potential source of information to track the HR, HRV, and other physiological observables. Self-reporting and sleep diaries could also be used to determine sleep and exercise levels for each individual.

## Environmental factors

The impacts of environmental factors on cardiac health are well reported (Basu 2009; Basu and Ostro 2008; Dilaveris et al. 2006). Both high and low temperature extremes are cardiovascular stressors that increase cardiovascular morbidity and mortality (Liu et al. 2015; Wolf et al. 2009; Basu 2009; Basu and Ostro 2008; Dilaveris et al. 2006). Acute and chronic air pollution exposure also induce adverse health effects via oxidative stress and inflammation, ultimately leading to increased cardiovascular mortality (Brook et al. 2004; Kim et al. 2021; Wang et al. 2020; Nuvolone et al. 2011). Despite the clinical and public-health significance of these factors, their absence in conventional cardiovascular modeling stems in large part from the practical challenges surrounding their implementation. In daily life, individuals move through numerous microenvironments and are exposed to continuous variations in temperatures and pollution levels. The impacts of these effects on different individuals are highly variable and complex, which creates particular challenges for systematically collecting and incorporating data into models.

### External temperature variations

The body’s overall response to temperature variations is inherently multifaceted, demanding a holistic approach to thermoregulation modeling. Deviations from homeostatic temperatures prompt an adaptive thermoregulatory response not only through the modulation of hemodynamic behavior via blood vessel dilation or constriction (Coccarelli et al. 2017), but also by stimulating additional regulatory mechanisms such as sweating (Fiala et al. 2001; Hirata et al. 2015) and shivering (Smith 1991). Further levels of complexity are required when considering the rheological and oxygen-transfer properties of blood flow (Secomb 2017; Coccarelli and Nelson 2023), heat transfer dynamics through different tissue types, and pathophysiological conditions (Coccarelli et al. 2017). The balance of all these interconnected mechanisms may vary significantly between individuals, with a particular sensitivity to age (Coccarelli et al. 2018).
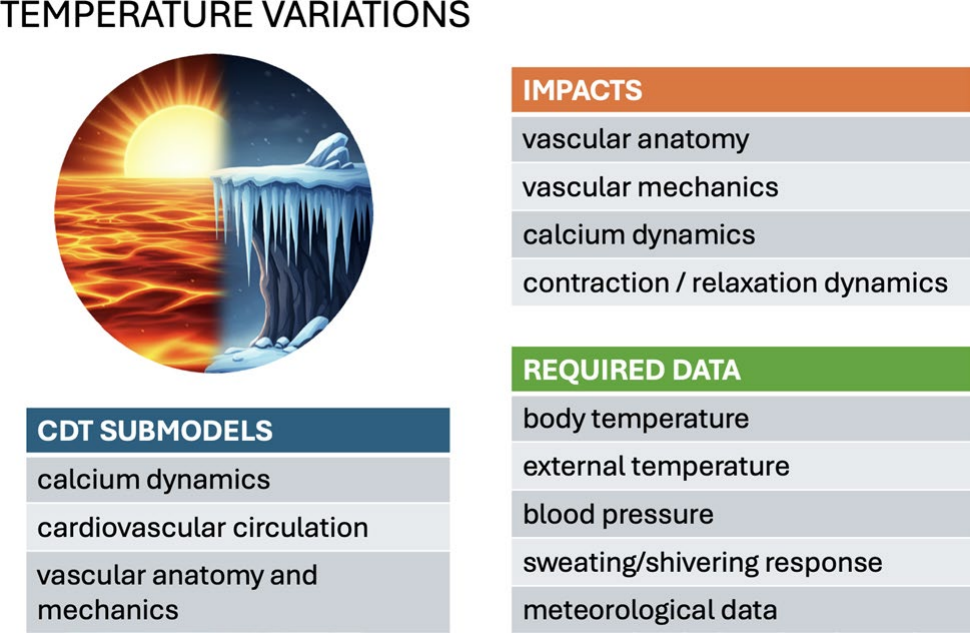


The heart reacts to changes in the body temperature at the cellular level. Katsnelson et al. used a mathematical model of myocardial contraction-relaxation regulation to investigate the effect of high temperatures (following temperature changes of the order of 24–37 $^\circ$C) on a range of factors, including an increase in calcium pumping into the sarcoplasmic reticulum, a slowing of the rise of the calcium transient, and an acceleration of cross-bridge cycling (Katsnelson et al. 2000). These cell-level effects reproduce a decrease in load-dependent relaxation and reduced sarcomere shortening, as observed clinically. The broader impact of such cellular-level responses to temperature on the whole-heart mechanics remains to be systematically explored in multi-scale simulations.

Temperature effects also occur at a higher systemic level. The main cardiovascular manifestations of acute environmental temperature variations relate to the vascular resistance via the arterial morphology, as the body seeks to control blood circulation to facilitate or hinder heat exchange with the surroundings. The biophysical model of Mynard and Smolich describes the body vasculature as a 1D system and the microvasculature using a 0D nonlinear Windkessel approximation (Mynard and Smolich 2015). The heart is represented as a lumped-parameter 0D entity with a time-varying elastance and a pressure-dependent source resistance. With reasonable accuracy, the model predicts characteristic clinically observed pressure and flow waveforms using wave-intensity analysis. This validation confirms the advantage of employing such approximations, compared to more detailed (and computationally demanding) 3D vasculature models. An explicit model of heat exchange with the environment by Coccarelli et al. describes the vasculature as a network of cylinders and elastic tubes (Coccarelli et al. 2017). This model was validated against measurements of the time to lethal hypothermia across a range of environmental temperatures.

To conclude, existing modeling frameworks demonstrate the robustness of the circulatory system’s thermoregulatory function. They lend themselves to simulating the body’s hyperemic response to variations in blood-flow demand within a clinical context, as reviewed by Coccarelli and Nelson (2023). Continuous data collection is likely to be an essential ingredient for the accurate modeling of the effects of external temperature variations on the cardiovascular system. This would require the systematic use of portable or wearable devices capable of monitoring changes in temperature, blood pressure, and physiological responses such as sweating and shivering.

### Pollution and contaminants

Atmospheric pollution, particularly in the form of particulate matter (with typical size of a few microns and with various compositions), has been linked to a range of cardiovascular diseases, including arrhythmia, atherosclerosis, hypertension, and myocardial infarction (Brook et al. 2004). Fine pollutant particles arguably contribute to atherosclerosis, microvascular occlusions, and the excessive production of reactive oxygen species, further impacting vascular dilation and arterial resistance (Shimokawa 1999; Haverich 2017). Radiation-induced cardiotoxicity, a known side effect of cancer radiotherapy, was recently investigated using mouse experimental models, detecting the deterioration of the cardiac tissue's contractility properties (Mukherjee et al. 2025).
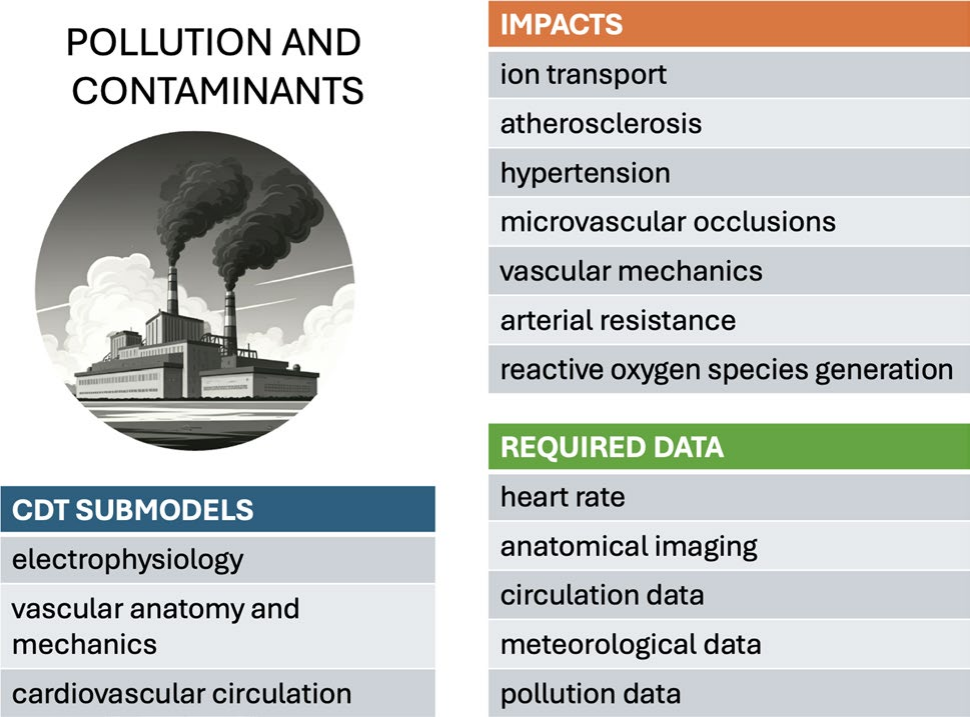


To our knowledge, a systematic biomechanical model of the cardiovascular system, explicitly in the context of response to pollution and contaminants, remains to be formulated. However, a range of multiphysics modeling studies have analyzed the initiation and development of atherosclerosis (El Khatib et al. 2007; Liu et al. 2010; Arturo and Lourdes 2018; Silva et al. 2020; Soleimani et al. 2021). These models highlight the interplay between arterial wall thickening, inflammatory processes, nutrient transport, and blood-flow dynamics.

Long-term exposure to pollutants could be integrated into existing cardiovascular risk models through studies that compare individuals with high and low exposure levels. Such studies would require accurate data on pollutant exposure, either through wearable sensors or lifestyle and routine self-reports, as well as a reliable measure of atherosclerosis or hypertension progression. While hypertension is relatively straightforward to monitor, assessing atherosclerosis progression would necessitate coronary CT angiography or coronary calcium scoring. However, both imaging methods involve radiation exposure, posing challenges for identifying suitable clinical trial populations, particularly for repeated measurements.

## Other stressors

Providing a comprehensive list of cardiac stressors was beyond the scope and capability of this review. Our aim, in the above selection, was to present a spectrum of concrete case examples of known stressors displaying various degrees of development in terms of biomechanical modeling within the CDT perspective. The list could be extended to include other stressors that, to greater or lesser extents, have also received attention in cardiovascular modeling studies, e.g., alcohol consumption (Sutanto et al. 2020), psychological stress [takotsubo cardiomyopathy (Land et al. 2014)], and adrenaline (Geoffrey 2010). Additional stressors, while being well documented clinically or experimentally, await the establishment of systematic mathematical representations, including food restriction, and dietary habits. Contributors meriting consideration within specific contexts include age-induced (Sessions and Engler 2016; Cuomo et al. 2017) or ethnicity-based variations (van Apeldoorn et al. 2024; Georgiopoulos et al. 2024; Parke et al. 2024), which may predispose population sub-groups to cardiovascular disease. Many of these stressors can have a direct impact on the cardiovascular system in isolation or via interactions with other external stressors.

Obesity, a notorious cardiovascular stressor, is associated with lower exercise capacity, increased blood pressure, increased cardiac output and increased resistance to blood flow in the peripheral skeletal system. To incorporate the effects of obesity into lumped-parameter models would involve adjusting parameters related to blood volume, vascular resistance, cardiac function, and potentially the autonomic nervous system. These changes would lead to simulations reflecting the hemodynamic and regulatory alterations observed in obese individuals, such as increased blood pressure, cardiac output, altered HRV, and increased cardiac workload. More sophisticated adaptations could involve incorporating the impact of metabolic factors on vascular properties over time.

Gravitation-induced stresses, while being implicitly present in the standard heart, may cause significant differential responses under extreme conditions of, e.g., space flight or space exploration. Gravity creates hydrostatic pressure gradients, influences venous return (requiring mechanisms like valves and muscle pump), causes fluid shifts and blood volume changes (especially in altered gravity), and can lead to long-term cardiac structural adaptations. Furthermore, the baroreceptor reflex is calibrated and validated against physiological responses observed under the influence of Earth’s gravity, including those elicited by postural changes that induce gravitational stress. Accurate models, particularly for space flight or clinical scenarios involving posture, must incorporate these gravitational influences on fluid dynamics, pressure control, and cardiac structure. Models investigating the effects of acceleration forces, homeostatic regulation, and ventilation-perfusion matching on the human cardiovascular system were reviewed by Morris et al. (2024).

## Discussion

The biomechanical modeling of the cardiac system is in large measure already a mature science. The past decades have seen a sustained development from basic descriptions of muscle contraction to large-scale computational simulations of whole-heart function. The exploitation of models as practical tools to aid clinical practice is becoming increasingly established, driven by our growing understanding of fundamental physiology, ever-increasing computational capacities, and the availability of unprecedently large patient databases (Rodero et al. 2023). Current technological developments are broadening the scope for application to new territories. The aim of this review was to illustrate this endeavor by highlighting a selection of promising avenues for further developing clinical applications of biomechanical models. These cases were selected for their importance in public health and their potential amenability to modeling within the context of CDTs. As summarized in Fig. 1, the present state of model development is highly variable. Nonetheless, although each case presents its own set of challenges, we can identify the three following general themes to guide further work.

### Challenge 1: Mathematical modeling frameworks

The first challenge is to obtain mathematical frameworks that adequately describe the physiological system and accommodate the stressors of interest. This challenge is compounded by the vast time scales of many stressor effects (months or years) compared to a single heart beat. This necessitates model development on several levels to ensure that physiological behavior is correctly captured on both short and long time scales.

Models must firstly simulate the correct physiological behavior on the time scale of a single beat. Many existing biomechanical models, focusing on this time scale, already provide handles for incorporating patient-specific variations, which may in turn be tuned to simulate specific stressors. Degrees of freedom in models include boundary conditions (e.g., circulatory system pressures), HRs, or electrophysiological model parameters. Model evolution and refinement is a continual process, balancing systematic mechanistic formulations with pragmatic phenomenological approximations. This progress relies predominantly on the elucidation of the relevant mechanistic pathways, revealed by persistent basic physiological research, before they can be realistically implemented within model structures. As indicated in this review, existing mathematical formalisms often provide building blocks that can be integrated into simulation frameworks, at least as a first approximation. Ultimately, a degree of model simplification and lumping of parameters is dictated by the system complexity and limitations in data availability. However, this also depends pragmatically on the scope and aims of the intended application.

Looking beyond the single-beat time scale, the physiological effect of daily stressors and clinical treatments often become manifested over time scales of months or years. This requires the development of models that simulate the growth and remodeling (G&R) of the heart and blood vessels and to predict long-term outcomes, guide treatment planning, and optimize therapeutic strategies. Such modeling endeavors are inherently computationally expensive, which may require a balance of mechanistic and phenomenological models. For instance, Yoshida et al. combined a phenomenological G&R model of the left ventricle with a hormonal-effects model during pregnancy (Yoshida et al. 2022). The hormone model was implemented within a systems-biology framework that was relatively cheap compared to the G&R simulations (or potential mechanics simulations). This opens the possibility to update the hormonal model, integrated within the G&R simulations, using new hormone measurements. Such model compartmentalization may become essential for integrating vast data flows to simulate months of growth on a time scale of minutes (Witzenburg and Holmes 2018). Ultimately, the choice of modeling style must again be decided based on the intended clinical application.

Physics-based approaches, such as constrained mixture models (Gebauer et al. 2023), provide a more mechanistic description of cardiac changes, albeit at a higher computational cost. A key benefit is the prediction of the long-term progression of the heart in terms of its volume, pressures, strains, or shape. However, updating the remodeling process typically requires data collected at the hospital, rather than at the patient’s home, which makes the inclusion of short- or medium-term effects (e.g., heart-rate variability or hormonal fluctuations) problematic. Ultimately, the choice of modeling style must again be decided based on the intended clinical application.

### Challenge 2: Data acquisition

A second challenge is to identify and collect relevant personalized data that allow the tuning of target model parameters. As the case studies presented above demonstrate, this calls for the exploitation of data sources not previously considered as part of standard clinical practice. Depending on the stressors being considered, these new data may range from real-time physiological data to meteorological, atmospheric, or satellite data. Despite the clear challenges this presents, the ongoing development of novel data-monitoring technologies is making this prospect increasingly realistic.

Wearable devices are commercially available or clinical remote monitors containing electronic gadgets (e.g., patches, handhelds, eyeglasses, wristwatches, fitness trackers) that enable the real-time tracking, analysis, or transmission of health-related data (Andrew Hughes et al. 2023). Wearable-derived physiological data can be combined with environmental sensors, accelerometers, and gyroscopes, and other types of user-specific inputs (e.g., mood or period tracking). Physiological sensors can be optical like photoplethysmography signals and pulse oximeters (Aymen 2014) for the HR, blood flow, and blood oxygen saturation; temperature sensors, or electrodes for ECG data (Furrukh et al. 2020). Patches are ECG-recording devices that contain electrodes fastened to the chest for the continuous ambulatory monitoring of a patient (Schreiber et al. 2014). Environmental sensors can include accelerometers, gyroscopes, GPS coordinates, altitude, and ambient temperature for activity and motion tracking. Some devices allow for user input for other factors like mood, menstrual cycle tracking, food and water intake. Environmental and physiological data are combined with software to provide estimates of metrics for fitness, physical performance, cardiac function (Gill et al. 2024; Bayoumy et al. 2021), or sleep. Finally, when in the context of a network of communications and a paired mobile device (smartphones), wearable devices can produce more sophisticated metrics like overall health scores, fitness age estimates and activity trends.

New research exploiting the versatility of portable technologies has been used for personalized risk assessment, remote patient management and clinical decision support. Patient-specific models based on machine-learning predictors are being used to predict adverse events like asymptomatic atrial fibrillation episodes (Aymen 2014; Andrew Hughes et al. 2023; Mohamoud et al. 2024) or to identify subtle changes in cardiac function for heart failure patients (Bayoumy et al. 2021; Mohamoud et al. 2024). Clinical devices have been used for clinical decision support, creating automated alters for significant changes in cardiac parameters (Alian 2014).

Despite significant advances in exploiting wearable device technology for cardiac monitoring, its direct integrability into computational simulations remains an emerging research field. An important caveat is that most commercially available devices, being designed for specific consumer applications, yield heavily processed (smoothed and filtered) data under proprietary models. The consequent loss of the raw data may preclude rigorous uncertainty quantification, which in turn may limit the reliability of simulations. The challenge is therefore to ensure that models are robust enough against signal intermittence, outliers, and unverified measurements.

### Challenge 3: Data integration

The third challenge concerns the systematic incorporation of data into the models. Various types of data assimilation methodologies have been developed to estimate the state of a complex system, typically based on a prior estimate and incoming observations (Asch et al. 2016). Variational methods provide computationally efficient algorithms for retrospectively minimizing discrepancies between model predictions and observations by an iterative process (Rohan and Cimrman 2002; Sermesant et al. 2006). Sequential approaches, on the other hand, are better suited to updating a system state as new observations become available. For example, ensemble Kalman filtering (EnKF) (Hoffman and Cherry 2020; Corrado et al. 2015) involves estimating the “most likely” state of a system based on a prior estimate and an ensemble of continuously updated model simulations that can be integrated in real time.

EnKF describes the uncertainty in the model state and parameters through an ensemble of “particles” (samples) that propagate in time following the model dynamics (i.e., a prediction functionality). As new data become available, the algorithm updates the state parameters by maximizing the likelihood of consistency between the model output and the data (analysis functionality). This estimation approximates the (posterior) probability distribution of the model parameters and hence provides an objective estimation of the model uncertainty based on the available data. EnKF does not require intrusive modifications of simulation software and allows parallelization over sample updates. It can be applied to sparse data and multiphysics modeling. Variants of the Kalman filtering approach have been developed to integrate multi-fidelity data (Habibi et al. 2021; Moireau et al. 2008).

Solving organ-scale computational models for the purpose of model calibration is often prohibitively expensive, particularly when simulations are required over long temporal horizons. This computational burden limits their practical application in time-sensitive or large-scale settings. Surrogate models offer a compelling solution in scenarios where the primary objective is to approximate the relationship between model parameters and specific outputs of interest. For instance, Gaussian process emulators have been used to capture the relationship between cellular-level behavior and clinical outcomes, a method likely to facilitate the calibration of patient-specific digital twins (Strocchi et al. 2023).

Model calibration frequently necessitates numerous evaluations of the computational model across varying parameter sets, further exacerbating computational demands. Machine-learning techniques can alleviate this challenge by learning the underlying parameter-to-output mappings. Surrogate models (including K-nearest neighbors (KNN), XGBoost, and multi-layer perceptron (MLP) regressors) have been used to predict clinical measurements (e.g., pressure and volume) from underlying model parameters (e.g., myocardial stiffness) (Cai et al. 2021). The full computational model was initially run on a sampled set of parameters to generate training data, after which the trained multi-layer models could rapidly predict outcomes for new parameter configurations.

However, patient-specific structural and anatomical variability often necessitates the construction of individualized surrogate models, potentially reducing the scalability and efficiency of this approach. To address this, a surrogate modeling strategy was proposed for estimating local activation times, accounting for anatomical variability, thereby offering a partial solution to the personalization challenge while preserving computational efficiency (Yin et al. 2024).

## Conclusion

CDT development requires model integration on manifold levels and the processing of many unknown parameters and interrelations. The aim of this review was to illustrate, through a selection of established cardiovascular stressors, the challenge of systematically assembling these multiple elements to form coherent models yielding reliable predictions. The inclusion of all possible stressors within a single modeling framework is inevitably an unattainable objective. Arguably, even when considering a particular stressor, the implementation and complexity of the corresponding digital twin may vary according to the requirements of its intended application, balancing physiological soundness, quantitative precision, and computational tractability. The value of CDTs, a concept borrowed from other fields of engineering, is perhaps best understood by viewing them as an ideal end goal that is approached through continually improved approximations.

The challenges of stressor implementation are compounded by the possibility of interactions between stressors, a dimension that is still largely unexplored. For example, future modeling efforts may consider stressor interactions such as the impact of body weight and dietary patterns on the response to exercise, or the modulation of cardiovascular behavior and properties by age (Sessions and Engler 2016; Cuomo et al. 2017) or ethnicity (van Apeldoorn et al. 2024; Georgiopoulos et al. 2024; Parke et al. 2024). A single model has limited predictive power in real-world situations, making a multi-model statistical ensemble description essential. This approach, allowing for a multi-level analysis while keeping track of the uncertainty added at each level, has been applied in vascular modeling through the efficient interfacing of multiple simulation environments (Huttary et al. 2017).

These challenges notwithstanding, the CDT concept is becoming increasingly present in the parlance of biomechanical modeling. Although the terminology itself reflects an aspiration that remains inevitably utopic, it expresses an appetite for exploiting the latest technical advances to extend the outreach of computational research into new territory. In particular, there is now greater scope for exploiting data acquired outside standard clinical settings to guide and assist in providing more individualized medical care. As the elements of CDT technology become progressively realized, they will be able to provide increasing valuable support to physicians when making decisions to achieve ever-improving standards of care for all cardiac patients.

## References

[CR1] Aguado-Sierra J, Brigham R, Baron AK, Gomez PD, Houzeaux G, Guerra JM, Carreras F, Filgueiras-Rama D, Vazquez M, Iaizzo PA, Iles TL, Butakoff C (2024) HPC framework for performing in silico trials using a 3D virtual human cardiac population as means to assess drug-induced arrhythmic risk. In: Heifetz A (ed) Methods in molecular biology, chapter High Perfo, vol 2716. Springer, Berlin 10.1007/978-1-0716-3449-3_14 37702946

[CR2] Alien AA, Shelley KH (2014) Photoplethysmography. Best Pract Res Clin Anaesthesiol 28(4):395–406 25480769 10.1016/j.bpa.2014.08.006

[CR3] Andrew Hughes Md, Shandhi MH, Master H, Dunn J, Brittain E (2023) Wearable devices in cardiovascular medicine. Circ Res 132(5):652–670 36862812 10.1161/CIRCRESAHA.122.322389 PMC9991078

[CR4] Arturo H, Lourdes T (2018) Numerical simulation of a porous medium-type atherosclerosis initiation model. Comput Fluids 169:380–387

[CR5] Asch M, Bocquet M, Nodet M (2016) Data assimilation

[CR6] Basu R (2009) High ambient temperature and mortality: a review of epidemiologic studies from 2001 to 2008. Environ Health 8(1):40 19758453 10.1186/1476-069X-8-40 PMC2759912

[CR7] Basu R, Ostro BD (2008) A multicounty analysis identifying the populations vulnerable to mortality associated with high ambient temperature in California. Am J Epidemiol 168(6):632–637 18663214 10.1093/aje/kwn170

[CR8] Bayoumy K, Gaber M, Elshafeey A, Mhaimeed O, Dineen EH, Marvel FA, Martin SS, Muse ED, Turakhia MP, Tarakji KG, Elshazly MB (2021) Smart wearable devices in cardiovascular care: where we are and how to move forward. Nat Rev Cardiol 18(8):581–599 33664502 10.1038/s41569-021-00522-7 PMC7931503

[CR9] Brook RD, Franklin B, Cascio W, Hong Y, Howard G, Lipsett M, Luepker R, Mittleman M, Samet J, Smith SC, Tager I (2004) Air pollution and cardiovascular disease: a statement for healthcare professionals from the expert panel on population and prevention science of the American Heart Association. Circulation 109(21):2655–2671 15173049 10.1161/01.CIR.0000128587.30041.C8

[CR10] Cai L, Ren L, Wang Y, Xie W, Zhu G, Gao H (2021) Surrogate models based on machine learning methods for parameter estimation of left ventricular myocardium. R Soc Open Sci. 10.1098/rsos.201121 10.1098/rsos.201121 PMC7890479 33614068

[CR11] Cappuccio FP, Cooper D, Delia L, Strazzullo P, Miller MA (2011) Sleep duration predicts cardiovascular outcomes: a systematic review and meta-analysis of prospective studies. Eur Heart J 32(12):1484–1492 21300732 10.1093/eurheartj/ehr007

[CR12] Cheng L, Khoo M (2012) Modeling the autonomic and metabolic effects of obstructive sleep APNEA: a simulation study. Front Physiol 2:1–20 10.3389/fphys.2011.00111 PMC3250672 22291654

[CR13] Cheng L, Ivanova O, Fan H-H, Khoo M (2010) An integrative model of respiratory and cardiovascular control in sleep-disordered breathing. Respir Physiol Neurobiol 174(1–2):4–28 20542148 10.1016/j.resp.2010.06.001 PMC2965826

[CR14] Cieniawa J, Baszak J, Olchowik G, Widomska J (2013) Modeling gender effects on electrical activity of single ventricular myocytes. Comput Biol Med 43(8):1063–1072 23726761 10.1016/j.compbiomed.2013.05.002

[CR15] Coccarelli A, Nelson MD (2023) Modeling reactive hyperemia to better understand and assess microvascular function: a review of techniques. Ann Biomed Eng 3(51):479–2167492 10.1007/s10439-022-03134-5 PMC9928923 36709231

[CR16] Coccarelli A, Boileau E, Parthimos D, Nithiarasu P (2017) Modelling accidental hypothermia effects on a human body under different pathophysiological conditions. Med Biol Eng Comput 55(12):2155–2167 28585067 10.1007/s11517-017-1657-3 PMC5680406

[CR17] Coccarelli A, Hasan HM, Carson J, Parthimos D, Nithiarasu P (2018) Influence of ageing on human body blood flow and heat transfer: a detailed computational modelling study. Int J Numer Methods Biomed Eng 34(10):e3120 10.1002/cnm.3120 PMC6220937 29932495

[CR18] Corrado C, Gerbeau JF, Moireau P (2015) Identification of weakly coupled multiphysics problems. Application to the inverse problem of electrocardiography. J Comput Phys 283:271–298

[CR19] Cuomo F, Roccabianca S, Desmond Dillon-Murphy, Xiao N, Humphrey JD, Alberto Figueroa C (2017) Effects of age-associated regional changes in human central artery mechanics on systemic hemodynamics revealed by computational modeling. PLoS ONE 12(3):1–21 10.1371/journal.pone.0173177 PMC5333881 28253335

[CR20] Darakjian LI, Kaddoumi A (2019) Physiologically based pharmacokinetic/pharmacodynamic model for caffeine disposition in pregnancy. Mol Pharm 16(3):1340–1349 30689395 10.1021/acs.molpharmaceut.8b01276

[CR21] Dilaveris P, Synetos A, Giannopoulos G, Gialafos E, Pantazis A, Stefanadis C (2006) CLimate Impacts on Myocardial infarction deaths in the Athens TErritory: the CLIMATE study. Heart 92(12):1747–1751 16840509 10.1136/hrt.2006.091884 PMC1861268

[CR22] Doris U, Kharche S, Petkova M, Borbas B, Logantha Sunil Jit RJ, Fedorenko O, Maczewski M, Mackiewicz U, Zhang Y, Chahal A, D’souza A, Atkinson AJ, Dobrzynski H, Yanni J (2019) A sexy approach to pacemaking: differences in function and molecular make up of the sinoatrial node. Histol Histopathol 34(11):1255–1268 30968943 10.14670/HH-18-115

[CR23] Dupuis LJ, Arts T, Prinzen FW, Delhaas T, Lumens J (2018) Linking cross-bridge cycling kinetics to response to cardiac resynchronization therapy: a multiscale modelling study. Europace 20:87–93 10.1093/europace/euy230 30476050

[CR24] Eisner DA, Caldwell JL, Kistamás K, Trafford AW (2017) Calcium and excitation–contraction coupling in the heart. Circul Res 121(2):181–195 10.1161/CIRCRESAHA.117.310230 PMC5497788 28684623

[CR25] Eisner DA, Greensmith DJ, Trafford AW (2024) Calcium flux balance in the heart: how many of the important questions are really unanswered? J Physiol 24:7103–7107 10.1113/JP287849 39526597

[CR26] El Khatib N, Génieys S, Volpert V (2007) Atherosclerosis initiation modeled as an inflammatory process. Math Model Nat Phenomena 2(2):126–141

[CR27] Elisa M, Ursino M (2002) Cardiovascular response to dynamic aerobic exercise: a mathematical model. Med Biol Eng Comput 40(6):660–674 12507317 10.1007/BF02345305

[CR28] Fendt R, Hofmann U, Schneider ARP, Schaeffeler E, Burghaus R, Yilmaz A, Blank LM, Kerb R, Lippert J, Schlender JF, Schwab M, Kuepfer L (2021) Data-driven personalization of a physiologically based pharmacokinetic model for caffeine: a systematic assessment. CPT: Pharmacom Syst Pharmacol 10(7):782–793 10.1002/psp4.12646 PMC8302243 34053199

[CR29] Fiala D, Lomas KJ, Stohrer M (2001) Computer prediction of human thermoregulatory and temperature responses to a wide range of environmental conditions. Int J Biometeorol 45:143–159 11594634 10.1007/s004840100099

[CR30] Fogli IA, Haibo N, Sicheng Z, Xianwei Z, Raffaele C, Chi YP, Uma S, Clancy Colleen E, Edwards Andrew G, Stefano M, Eleonora G (2021) Sex-specific classification of drug-induced Torsade de Pointes susceptibility using cardiac simulations and machine learning. Clin Pharmacol Ther 110(2):380–391 33772748 10.1002/cpt.2240 PMC8316283

[CR31] Furrukh S, Isselbacher EM, Singh JP, Kevin HE, Bhupesh P, Armoundas AA (2020) Wearable devices for ambulatory cardiac monitoring: JACC state-of-the-art review. J Am Coll Cardiol 75(13):1582–1592 32241375 10.1016/j.jacc.2020.01.046 PMC7316129

[CR32] Gaborit N, Varro A, Le Bouter S, Szuts V, Escande D, Nattel S, Demolombe S (2010) Gender-related differences in ion-channel and transporter subunit expression in non-diseased human hearts. J Mol Cell Cardiol 49(4):639–646 20600101 10.1016/j.yjmcc.2010.06.005

[CR33] Gao Z, Xiong J, Chen Z, Deng X, Zaipin X, Sun A, Fan Y (2020) Gender differences of morphological and hemodynamic characteristics of abdominal aortic aneurysm. Biol Sex Differ 11(1):1–10 32693818 10.1186/s13293-020-00318-3 PMC7372899

[CR34] Gebauer AM, Pfaller MR, Braeu FA, Cyron CJ, Wall WA (2023) A homogenized constrained mixture model of cardiac growth and remodeling: analyzing mechanobiological stability and reversal. Biomech Model Mechanobiol 22(6):1983–2002 37482576 10.1007/s10237-023-01747-w PMC10613155

[CR35] Geoffrey CJ (2010) Model-based prediction of the patient-specific response to adrenaline. Open Med Inform J 4(1):149–163 21603091 10.2174/1874431101004010149 PMC3098554

[CR36] Georgiopoulos G, Faconti L, Mohamed AT, Figliozzi S, Asher C, Keehn L, McNally R, Alfakih K, Vennin S, Chiribiri A, Lamata P, Chowienczyk P, Masci P-G (2024) Cardiac remodelling patterns in hypertension: does ethnicity matter? Eur Heart J Cardiovasc Imaging 25(7):912–913 38597630 10.1093/ehjci/jeae097 PMC11210986

[CR37] Gerach T, Schuler S, Fröhlich J, Lindner L, Kovacheva E, Moss R, Wülfers EM, Seemann G, Wieners C, Loewe A (2021) Electro-mechanical whole-heart digital twins: a fully coupled multi-physics approach. Mathematics 9(11):1247

[CR38] Gerach T, Appel S, Wilczek J, Golba KS, Jadczyk T, Loewe A (2022) Dyssynchronous left ventricular activation is insufficient for the breakdown of wringing rotation. Front Physiol 13(May):1–11 10.3389/fphys.2022.838038 PMC9124904 35615669

[CR39] Gerach T, Schuler S, Wachter A, Loewe A (2023) The impact of standard ablation strategies for atrial fibrillation on cardiovascular performance in a four-chamber heart model. Cardiovasc Eng Technol 14(2):296–314 36652165 10.1007/s13239-022-00651-1 PMC10102113

[CR40] Gill SK, Barsky A, Guan X, Bunting KV, Karwath A, Tica O, Stanbury M, Haynes S, Folarin A, Dobson R, Kurps J, Asselbergs FW, Grobbee DE, Camm AJ, Eijkemans M, Gkoutos GV, Kotecha D (2024) Consumer wearable devices for evaluation of heart rate control using digoxin versus beta-blockers: the RATE-AF randomized trial. Nat Med. 10.1038/s41591-024-03094-4 10.1038/s41591-024-03094-4 PMC11271403 39009776

[CR41] Gonzalez-Martin P, Sacco F, Butakoff C, Doste R, Bederian C, Gutierrez-Espinosa-de-los-Monteros-Lilian K, Houzeaux G, Iaizzo PA, Iles TL, Vazquez M, Aguado-Sierra J (2023) Ventricular anatomical complexity and sex differences impact predictions from electrophysiological computational models. PLoS ONE 18(February):1–25 10.1371/journal.pone.0263639 PMC9925004 36780442

[CR42] Govindarajan V, Kolanjiyil A, Wanna C, Kim H, Prakash S, Chandran KB, McPherson DD, Johnson NP (2024) Biomechanical evaluation of aortic valve stenosis by means of a virtual stress test: a fluid–structure interaction study. Ann Biomed Eng 52(2):414–424 37957528 10.1007/s10439-023-03389-6

[CR43] Granegger M, Schweiger M, Daners MS, Meboldt M, Hübler M (2018) Cavopulmonary mechanical circulatory support in Fontan patients and the need for physiologic control: a computational study with a closed-loop exercise model. Int J Artif Organs 41(5):261–268 29521133 10.1177/0391398818762359

[CR44] Grissinger M (2010) The five rights: a destination without a map. Pharm Therapeut 35(10):542

[CR45] Habibi M, D’Souza RM, Dawson Scott TM, Arzani A (2021) Integrating multi-fidelity blood flow data with reduced-order data assimilation. Comput Biol Med 135:104566 34157468 10.1016/j.compbiomed.2021.104566

[CR46] Haverich A (2017) A surgeon’s view on the pathogenesis of atherosclerosis. Circulation 135(3):205–207 28093492 10.1161/CIRCULATIONAHA.116.025407

[CR47] Hellgren KT, Ni H, Morotti S, Grandi E (2023) Predictive male-to-female translation of cardiac electrophysiological response to drugs. JACC: Clin Electrophysiol 9(12):2642–2648 37768254 10.1016/j.jacep.2023.08.016 PMC11390274

[CR48] Hirata A, Nomura T, Laakso I (2015) Computational estimation of body temperature and sweating in the aged during passive heat exposure. Int J Therm Sci 89:154–163

[CR49] Hoffman MJ, Cherry EM (2020) Sensitivity of a data-assimilation system for reconstructing three-dimensional cardiac electrical dynamics: sensitivity of data-assimilation system. Philos Trans R Soc A Math Phys Eng Sci 378(2173):1. 10.1098/rsta.2019.0388 10.1098/rsta.2019.0388 PMC7287341 32448069

[CR50] Hunter S, Robson SC (1992) Adaptation of the maternal heart in pregnancy. Heart 68(12):540–543 10.1136/hrt.68.12.540 PMC1025680 1467047

[CR51] Huttary R, Goubergrits L, Schütte C, Bernhard S (2017) Simulation, identification and statistical variation in cardiovascular analysis (SISCA)—a software framework for multi-compartment lumped modeling. Comput Biol Med 87:104–123 28577434 10.1016/j.compbiomed.2017.05.021

[CR52] Kanki M, Nath AP, Xiang R, Yiallourou S, Fuller PJ, Cole TJ, Cánovas R, Young MJ (2023) Poor sleep and shift work associate with increased blood pressure and inflammation in UK Biobank participants. Nat Commun 14(1):1–15 37925459 10.1038/s41467-023-42758-6 PMC10625529

[CR53] Katsnelson LB, Markhasin VS, Khazieva NS (2000) Mathematical modeling of the effect of the sarcoplasmic reticulum calcium pump function on load dependent myocardial relaxation. Gen Physiol Biophys 19(2):137–170 11156439

[CR54] Kaye DM, Wolsk E, Nanayakkara S, Mariani J, Hassager C, Gustafsson F, Moller JE, Sunagawa K, Burkhoff D (2021) Comprehensive physiological modeling provides novel insights into heart failure with preserved ejection fraction physiology. J Am Heart Assoc 10(19):e021584 34569288 10.1161/JAHA.121.021584 PMC8649144

[CR55] Kim SY, Kim SH, Wee JH, Min C, Han SM, Kim S, Choi HG (2021) Short and long term exposure to air pollution increases the risk of ischemic heart disease. Sci Rep 11(1):1–11 33658616 10.1038/s41598-021-84587-x PMC7930275

[CR56] Kurata Y, Hisatome I, Imanishi S, Shibamoto T (2002) Dynamical description of sinoatrial node pacemaking: improved mathematical model for primary pacemaker cell. Am J Physiol Heart Circul Physiol 283(52–5):2074–2101 10.1152/ajpheart.00900.2001 12384487

[CR57] Land S, Niederer S, Louch WE, Røe AT, Aronsen JM, Stuckey DJ, Sikkel MB, Tranter MH, Lyon AR, Harding SE, Smith NP (2014) Computational modeling of Takotsubo cardiomyopathy: effect of spatially varying beta-adrenergic stimulation in the rat left ventricle. Am J Physiol Heart Circul Physiol 307(10):H1487-96 10.1152/ajpheart.00443.2014 PMC4233305 25239804

[CR58] Lee A, O’Regan DP, Gould J, Sidhu B, Sieniewicz B, Plank G, Warriner DR, Lamata P, Rinaldi CA, Niederer S (2019) Sex-dependent QRS guidelines for cardiac resynchronization therapy using computer model predictions. Biophys J 117(12):2375–2381 31547974 10.1016/j.bpj.2019.08.025 PMC6990372

[CR59] Liu L, Wang W, Meng X, Gao J, Haiying W, Wang P, Weichun W, Wang L, Ma L, Zhang W (2010) Left ventricular hypertrophy induced by abdominal aortic banding and its prevention by angiotensin receptor blocker telmisartan—a proteomic analysis. J Physiol Biochem 66:329–338 20697985 10.1007/s13105-010-0039-1

[CR60] Liu C, Yavar Z, Sun Q (2015) Cardiovascular response to thermoregulatory challenges. Am J Physiol-Heart Circul Physiol 309(11):H1793–H1812 10.1152/ajpheart.00199.2015 PMC4698386 26432837

[CR61] Llopis-Lorente J, Baroudi S, Koloskoff K, Mora MT, Basset M, Romero L, Benito S, Dayan F, Saiz J, Trenor B (2023) Combining pharmacokinetic and electrophysiological models for early prediction of drug-induced arrhythmogenicity. Comput Methods Programs Biomed. 10.1016/j.cmpb.2023.107860 10.1016/j.cmpb.2023.107860 37844488

[CR62] Lumens J, Steve FCP, Walmsley J, Yim D, Manlhiot C, Dragulescu A, Grosse-Wortmann L, Mertens L, Prinzen FW, Delhaas T, Friedberg MK (2019) Relative impact of right ventricular electromechanical dyssynchrony versus pulmonary regurgitation on right ventricular dysfunction and exercise intolerance in patients after repair of tetralogy of fallot. J Am Heart Assoc 8(2) 10.1161/JAHA.118.010903 PMC6497336 30651018

[CR63] Lyon AR, Dent S, Stanway S, Earl H, Brezden-Masley C, Cohen-Solal A, Tocchetti CG, Moslehi JJ, Groarke JD, Bergler-Klein J, Khoo V, Tan LL, Anker MS, von Haehling S, Maack C, Pudil R, Barac A, Thavendiranathan P, Bonnie K, Neilan TG, Belenkov Y, Rosen SD, Iakobishvili Z, Sverdlov AL, Hajjar LA, Macedo A, Manisty C, Ciardiello F, Farmakis D, de Boer RA, Skouri H, Suter TM, Cardinale D, Witteles RM, Fradley MG, Herrmann J, Cornell RF, Wechelaker A, Mauro MJ, Milojkovic D, de Lavallade H, Ruschitzka F, Coats A, Seferovic PM, Chioncel O, Thum T, Bauersachs J, Andres MS, Wright DJ, López-Fernández T, Plummer C, Lenihan D (2020) Baseline cardiovascular risk assessment in cancer patients scheduled to receive cardiotoxic cancer therapies: a position statement and new risk assessment tools from the Cardio-Oncology Study Group of the Heart Failure Association of the European Society. Eur J Heart Fail 22(11):1945–1960 32463967 10.1002/ejhf.1920 PMC8019326

[CR64] Maltsev VA, Lakatta EG (2009) Synergism of coupled subsarcolemmal Ca2+ clocks and sarcolemmal voltage clocks confers robust and flexible pacemaker function in a novel pacemaker cell model. Am J Physiol Heart Circul Physiol 296(3):594–615 10.1152/ajpheart.01118.2008 PMC2660239 19136600

[CR65] Marin JM, Carrizo SJ, Vicente E, Agusti A (2005) Long-term cardiovascular outcomes in men with obstructive sleep apnoea–hypopnoea with or without treatment with continuous positive airway pressure: an observational study. Lancet 365(9464):1046–1053 15781100 10.1016/S0140-6736(05)71141-7

[CR66] Miller CE, Jordan JH, Thomas A, Weis JA (2021) Developing a biomechanical model-based elasticity imaging method for assessing hormone receptor positive breast cancer treatment-related myocardial stiffness changes. J Med Imaging 8(05):1–16 10.1117/1.JMI.8.5.056002 PMC8482312 34604442

[CR67] Mohamoud A, Jensen J, Buda KG (2024) Consumer-grade wearable cardiac monitors: What they do well, and what needs work. Clevel Clin J Med 91(1):23–29 10.3949/ccjm.91a.23030 38167395

[CR68] Moireau P, Chapelle D, Le Tallec P (2008) Joint state and parameter estimation for distributed mechanical systems. Comput Methods Appl Mech Eng 197(6–8):659–677

[CR69] Morotti S, Edwards AG, Mcculloch AD, Bers DM, Grandi E (2014) A novel computational model of mouse myocyte electrophysiology to assess the synergy between Na+ loading and CaMKII. J Physiol 592(6):1181–1197 24421356 10.1113/jphysiol.2013.266676 PMC3961080

[CR70] Morris PD, Anderton RA, Marshall-Goebel K, Britton JK, Lee S, Smith NP, van de Vosse FN, Ong KM, Newman TA, Taylor DJ, Chico T, Gunn JP, Narracott AJ, Hose DR, Halliday I (2024) Computational modelling of cardiovascular pathophysiology to risk stratify commercial spaceflight. Nat Rev Cardiol 21(667):681 10.1038/s41569-024-01047-5 39030270

[CR71] Moss R, Wülfers EM, Schuler S, Loewe A, Seemann G (2021) A fully-coupled electro-mechanical whole-heart computational model: influence of cardiac contraction on the ECG. Front Physiol. 10.3389/fphys.2021.778872 10.3389/fphys.2021.778872 PMC8716847 34975532

[CR72] Mukherjee T, Elliott S, Manikandan N, Higgins T, Zhong Y, Montalvo S, Saha D, Wansapura J, Avazmohammadi R, Alluri P (2025) Principal strain analysis for early detection of radiation-induced cardiotoxicity in a mouse model. Int J Radiat Oncol Biol Phys (**in press**) 10.1016/j.ijrobp.2025.03.029 PMC13474174 40174647

[CR73] Mynard JP, Smolich JJ (2015) One-dimensional haemodynamic modeling and wave dynamics in the entire adult circulation. Ann Biomed Eng 43(6):1443–1460 25832485 10.1007/s10439-015-1313-8

[CR74] NASEM (2024) Foundational research gaps and future directions for digital twins. National Academies Press, Washington 39088664

[CR75] Nguyen TD, Kadri OE, Voronov RS (2020) An introductory overview of image-based computational modeling in personalized cardiovascular medicine 10.3389/fbioe.2020.529365 PMC7546862 33102452

[CR76] Niederer S, Campbell KS, Campbell SG (2019) A short history of the development of mathematical models of cardiac mechanics. J Mol Cell Cardiol 127(2018):11–19 30503754 10.1016/j.yjmcc.2018.11.015 PMC6525149

[CR77] Nuvolone D, Balzi D, Chini M, Scala D, Giovannini F, Barchielli A (2011) Short-term association between ambient air pollution and risk of hospitalization for acute myocardial infarction: results of the cardiovascular risk and air pollution in Tuscany (RISCAT) study. Am J Epidemiol 174(1):63–71 21597098 10.1093/aje/kwr046

[CR78] Olsen NT, Goransson C, Vejlstrup N, Carlsen J (2021) Myocardial adaptation and exercise performance in patients with pulmonary arterial hypertension assessed with patient-specific computer simulations. Am J Physiol Heart Circul Physiol 321(5):H865–H880 10.1152/ajpheart.00442.2021 34448636

[CR79] Parke KS, Brady EM, Alfuhied A, Motiwale RS, Razieh CS, Singh A, Arnold JR, Graham-Brown M, Bilak JM, Ayton SL, Dattani A, Yeo JL, McCann GP, Gulsin GS (2024) Ethnic differences in cardiac structure and function assessed by MRI in healthy South Asian and White European people: a UK biobank study. J Cardiovasc Magn Reson 26(1):100001 38218434 10.1016/j.jocmr.2023.100001 PMC11211094

[CR80] Pearce NF, Kim E (2023) A new synergistic model for simulating exercise incorporating control mechanisms at cellular and organ scales. Comput Biol Med 163(May):107141 37327758 10.1016/j.compbiomed.2023.107141

[CR81] Peirlinck M, Costabal FS, Kuhl E (2021) Sex differences in drug-induced arrhythmogenesis. Front Physiol 12(August):1–25 10.3389/fphys.2021.708435 PMC8417068 34489728

[CR82] Pierre SR, St Peirlinck M, Kuhl E (2022) Sex matters: a comprehensive comparison of female and male hearts. Front Physiol 13(March):1–19 10.3389/fphys.2022.831179 PMC8980481 35392369

[CR83] Poppas A, Shroff SG, Korcarz CE, Hibbard JU, Berger DS, Lindheimer MD, Lang RM (1997) Serial assessment of the cardiovascular system in normal pregnancy: role of arterial compliance and pulsatile arterial load. Circulation 95(10):2407–2415 9170404 10.1161/01.cir.95.10.2407

[CR84] Pritchard JA (1965) Changes in the blood volume during pregnancy and delivery. Anesthesiology 26:393–399 14313451 10.1097/00000542-196507000-00004

[CR85] Rodero C, Baptiste T, Barrows RK, Lewalle A, Niederer S, Strocchi M (2023) Advancing clinical translation of cardiac biomechanics models: a comprehensive review, applications and future pathways. Front Phys 11(November):1–23 10.3389/fphy.2023.1306210 PMC7615748 38500690

[CR86] Roel M, Rijks JHJ, Beela AS, Bressi E, Grieco D, Delhaas T, Luermans JGLM, Prinzen FW, Vernooy K, Lumens J (2023) Comparison of novel ventricular pacing strategies using an electro-mechanical simulation platform. Europace 25(6):1–11 37306315 10.1093/europace/euad144 PMC10259067

[CR87] Rohan E, Cimrman R (2002) Sensitivity analysis and material identification for activated smooth muscle. Comput Assist Mech Eng Sci 9:519–541

[CR88] Sarmiento CA, Hernández AM, Serna LY, Mañanas MA (2021) An integrated mathematical model of the cardiovascular and respiratory response to exercise: model-building and comparison with reported models. Am J Physiol Heart Circul Physiol 320(4):H1235–H1260 10.1152/ajpheart.00074.2020 33416450

[CR89] Schreiber D, Sattar A, Drigalla D, Higgins S (2014) Ambulatory cardiac monitoring for discharged emergency department patients with possible cardiac arrhythmias. West J Emerg Med 15(2):194–198 24672611 10.5811/westjem.2013.11.18973 PMC3966438

[CR90] Secomb TW (2017) Blood flow in the microcirculation. Ann Rev Fluid Mech 49:443–461

[CR91] Sedaghati F, Gleason RL (2023) A mathematical model of vascular and hemodynamics changes in early and late forms of preeclampsia. Physiol Rep 11(8):1–21 10.14814/phy2.15661 PMC10132946 37186372

[CR92] Sermesant M, Moireau P, Camara O, Sainte-Marie J, Andriantsimiavona R, Cimrman R, Hill D, Chapelle D, Razavi R (2006) Cardiac function estimation from MRI using a heart model and data assimilation: advances and difficulties. Med Image Anal 10(4):642–656 16765630 10.1016/j.media.2006.04.002

[CR93] Sessions AO, Engler AJ (2016) Mechanical regulation of cardiac aging in model systems. Circ Res 118(10):1553–1562 27174949 10.1161/CIRCRESAHA.116.307472 PMC4868502

[CR94] Severi S, Fantini M, Charawi LA, Difrancesco D (2012) An updated computational model of rabbit sinoatrial action potential to investigate the mechanisms of heart rate modulation. J Physiol 590(18):4483–4499 22711956 10.1113/jphysiol.2012.229435 PMC3477753

[CR95] Shimokawa H (1999) Primary endothelial dysfunction: atherosclerosis. J Mol Cell Cardiol 31(1):23–37 10072713 10.1006/jmcc.1998.0841

[CR96] Silva T, Jäger W, Neuss-Radu M, Sequeira A (2020) Modeling of the early stage of atherosclerosis with emphasis on the regulation of the endothelial permeability. J Theor Biol 496:110229 32259543 10.1016/j.jtbi.2020.110229

[CR97] Smith CE (1991) A transient, three-dimensional model of the human thermal system. PhD thesis

[CR98] Soleimani M, Haverich A, Wriggers P (2021) Mathematical modeling and numerical simulation of atherosclerosis based on a novel surgeon’s view. Arch Comput Methods Eng 28(6):4263–4282 34257506 10.1007/s11831-021-09623-5 PMC8266171

[CR99] Strocchi M, Augustin CM, Gsell Matthias AF, Karabelas E, Neic A, Gillette K, Razeghi O, Prassl AJ, Vigmond EJ, Behar JM, Gould JS, Sidhu B, Rinaldi CA, Bishop MJ, Plank G, Niederer S (2020) A publicly available virtual cohort of four-chamber heart meshes for cardiac electro-mechanics simulations. PLoS ONE 15(6):e0235145 32589679 10.1371/journal.pone.0235145 PMC7319311

[CR100] Strocchi M, Longobardi S, Augustin CM, Gsell Matthias AF, Petras A, Rinaldi CA, Vigmond EJ, Plank G, Oates CJ, Wilkinson RD, Niederer S (2023) Cell to whole organ global sensitivity analysis on a four-chamber heart electromechanics model using Gaussian processes emulators. PLoS Comput Biol 19(6):e1011257 37363928 10.1371/journal.pcbi.1011257 PMC10328347

[CR101] Sturgess VE, Tune JD, Figueroa CA, Carlson BE, Beard DA (2024) Integrated modeling and simulation of recruitment of myocardial perfusion and oxygen delivery in exercise. J Mol Cell Cardiol 192(February):94–108 38754551 10.1016/j.yjmcc.2024.05.006

[CR102] Sutanto H, Cluitmans Matthijs JM, Dobrev D, Volders P, Bébarová M, Heijman J (2020) Acute effects of alcohol on cardiac electrophysiology and arrhythmogenesis: insights from multiscale in silico analyses. J Mol Cell Cardiol 146(May):69–83 32710981 10.1016/j.yjmcc.2020.07.007

[CR103] TeBay C, McArthur JR, Mangala M, Kerr N, Heitmann S, Perry MD, Windley MJ, Vandenberg Jamie I, Hill AP (2022) Pathophysiological metabolic changes associated with disease modify the proarrhythmic risk profile of drugs with potential to prolong repolarisation. Br J Pharmacol 179:2631–2646 34837219 10.1111/bph.15757

[CR104] Terrar DA (2024) Calcium flux balance across cell membranes in the heart: important unanswered questions with implications for the role of ryanodine receptors. J Physiol 19:4687–4691 10.1113/JP287223 39097828

[CR105] Trayanova N (2011) Whole-heart modeling: applications to cardiac electrophysiology and electromechanics. Circ Res 108(1):113–128 21212393 10.1161/CIRCRESAHA.110.223610 PMC3031963

[CR106] Trayanova N, Rice JJ (2011) Cardiac electromechanical models: from cell to organ. Front Physiol 2(August):1–19 21886622 10.3389/fphys.2011.00043 PMC3154390

[CR107] Tulchinsky D, Hobel CJ, Yeager E, Marshall JR (1972) Plasma estrone, estradiol, estriol, progesterone, and 17-hydroxyprogesterone in human pregnancy: I.Normal pregnancy. Am J Obstetr Gynecol 112(8):1095–1100 10.1016/0002-9378(72)90185-8 5025870

[CR108] Turnbull D, Rodricks JV, Mariano GF, Chowdhury F (2017) Caffeine and cardiovascular health. Regul Toxicol Pharmacol 89:165–185 28756014 10.1016/j.yrtph.2017.07.025

[CR109] Ursino M (1998) Interaction between carotid baroregulation and the pulsating heart: a mathematical model. Am J Physiol Heart Circul Physiol 275(44–5):1733–1747 10.1152/ajpheart.1998.275.5.H1733 9815081

[CR110] van Apeldoorn J, Hageman S, Harskamp RE, Agyemang C, van den Born Bert JH, van Dalen JW, Galenkamp H, Hoevenaar-Blom MP, Richard E, van Valkengoed IGM, Visseren FLJ, Dorresteijn JAN, van Charante EPM (2024) Adding ethnicity to cardiovascular risk prediction: external validation and model updating of SCORE2 using data from the HELIUS population cohort. Int J Cardiol 417:132525 39244095 10.1016/j.ijcard.2024.132525

[CR111] Varshneya M, Irurzun-Arana I, Campana C, Dariolli R, Gutierrez A, Pullinger TK, Sobie EA (2021) Investigational treatments for COVID-19 may increase ventricular arrhythmia risk through drug interactions. CPT: Pharmacomet Syst Pharmacol 10(2):100–107 10.1002/psp4.12573 PMC7753424 33205613

[CR112] Verkerk AO, Wilders R, Veldkamp MW, de Geringel W, Kirkels JH, Tan HL (2005) Gender disparities in cardiac cellular electrophysiology and arrhythmia susceptibility in human failing ventricular myocytes. Int Heart J 46(6):1105–1118 16394606 10.1536/ihj.46.1105

[CR113] Verkerk AO, Wilders R, Tan HL (2007) Gender disparities in torsade de pointes ventricular tachycardia. Neth Hear J 15(12):405–411 10.1007/BF03086040 PMC2213447 18239736

[CR114] Voskoboinik A, Kalman JM, Kistler PM (2018) Caffeine and arrhythmias: time to grind the data. JACC: Clin Electrophysiol 4(4):425–432 30067480 10.1016/j.jacep.2018.01.012

[CR115] Wang VY, Nielsen P, Nash MP (2015) Image-based predictive modeling of heart mechanics. Annu Rev Biomed Eng 17:351–383 26643023 10.1146/annurev-bioeng-071114-040609

[CR116] Wang B, Eum KD, Kazemiparkouhi F, Li C, Manjourides J, Pavlu V, Suh H (2020) The impact of long-term PM2.5 exposure on specific causes of death: exposure-response curves and effect modification among 53 million U.S. Medicare beneficiaries. Environ Health A Global Access Science 19(1):20 10.1186/s12940-020-00575-0 PMC7026980 32066433

[CR117] Witzenburg CM, Holmes JW (2018) Predicting the time course of ventricular dilation and thickening using a rapid compartmental model. J Cardiovasc Transl Res 11(2):109–122 29550925 10.1007/s12265-018-9793-1 PMC6546110

[CR118] Wolf K, Schneider A, Breitner S, von Klot S, Meisinger C, Cyrys J, Hymer H, Wichmann HE, Peters A (2009) Air temperature and the occurrence of myocardial infarction in Augsburg, Germany. Circulation 120(9):735–742 19687361 10.1161/CIRCULATIONAHA.108.815860

[CR119] Yang PC, Clancy CE (2012) In silico prediction of sex-based differences in human susceptibility to cardiac ventricular tachyarrhythmias. Front Physiol 3(September):1–12 23049511 10.3389/fphys.2012.00360 PMC3442371

[CR120] Yang PC, Kurokawa J, Furukawa T, Clancy CE (2010) Acute effects of sex steroid hormones on susceptibility to cardiac arrhythmias: a simulation study. PLoS Comput Biol 6(1):e1000658 20126530 10.1371/journal.pcbi.1000658 PMC2813260

[CR121] Yang PC, Perissinotti LL, López-Redondo F, Wang Y, DeMarco KR, Jeng MT, Vorobyov I, Harvey RD, Kurokawa J, Noskov SY, Clancy CE (2017) A multiscale computational modelling approach predicts mechanisms of female sex risk in the setting of arousal-induced arrhythmias. J Physiol 595(14):4695–4723 28516454 10.1113/JP273142 PMC5509858

[CR122] Yaniv Y, Stern MD, Lakatta EG, Maltsev VA (2013) Mechanisms of beat-to-beat regulation of cardiac pacemaker cell function by Ca2+ cycling dynamics. Biophys J 105(7):1551–1561 24094396 10.1016/j.bpj.2013.08.024 PMC3791306

[CR123] Yao J, Chen S, Guccione JM (2022) A Computationally efficient approach to simulate heart rate effects using a whole human heart model. Bioengineering 9(8):1–12 10.3390/bioengineering9080334 PMC9331290 35892747

[CR124] Yiling F, Jaume C-F, van den Boomen M, Joan HK, Shi C, Eder RA, Roche ET, Nguyen CT (2021) Characterization of exercise-induced myocardium growth using finite element modeling and Bayesian optimization. Front Physiol 12(August):1–14 10.3389/fphys.2021.694940 PMC8381603 34434115

[CR125] Yin M, Charon N, Brody R, Lu L, Trayanova N, Maggioni M (2024) A scalable framework for learning the geometry-dependent solution operators of partial differential equations. Nat Computat Sci 4(12):928–940 10.1038/s43588-024-00732-2 PMC11659174 39653845

[CR126] Yoshida K, Saucerman JJ, Holmes JW (2022) Multiscale model of heart growth during pregnancy: integrating mechanical and hormonal signaling. Biomech Model Mechanobiol 21(4):1267–1283 35668305 10.1007/s10237-022-01589-y

[CR127] Zhang X, Wu Y, Smith C, Louch WE, Morotti S, Dobrev D, Grandi E, Ni H (2024) Enhanced Ca2+-driven arrhythmias in female patients with atrial fibrillation: insights from computational Modeling. bioRxiv 10.1016/j.jacep.2024.07.020 PMC11602355 39340505

[CR128] Zulli A, Smith RM, Kubatka P, Novak J, Uehara Y, Loftus H, Qaradakhi T, Pohanka M, Kobyliak N, Zagatina A, Klimas J, Hayes A, Rocca GL, Soucek M, Kruzliak P (2016) Caffeine and cardiovascular diseases: critical review of current research. Eur J Nutr 55(4):1331–1343 26932503 10.1007/s00394-016-1179-z

